# Thickness changes in the corneal epithelium and Bowman’s layer after overnight wear of silicone hydrogel contact lenses

**DOI:** 10.1186/s12886-018-0956-2

**Published:** 2018-11-03

**Authors:** Fan Lu, Aizhu Tao, Weiwei Tao, Xiran Zhuang, Meixiao Shen

**Affiliations:** 1 School of Ophthalmology and Optometry, Wenzhou Medical University, 270 Xueyuan Road, Wenzhou, Zhejiang, 325027 China; 2 The First Affiliated Hospital of Wenzhou Medical University, Wenzhou, Zhejiang, China

**Keywords:** Thickness, Epithelium, Bowman’s layer, Overnight, Contact lens

## Abstract

**Background:**

To investigate thickness changes in the corneal epithelium and Bowman’s layer after overnight silicone hydrogel contact lens (CL) wear by using ultra-high resolution optical coherence tomography (UHROCT).

**Methods:**

Eleven subjects without CL wearing history were recruited for this study. An UHROCT was used to measure the thickness of the epithelium (ET), Bowman’s layer (BT), stroma (ST), and total cornea (CCT) at the center of both eyes. A silicone hydrogel CL was inserted in the right eye of each subject, and the fellow non-CL wearing left eye served as the control. The lens was inserted at 9:30 pm and removed at 8:00 am the next morning. The subjects were evaluated at 9:00 pm (baseline), 9:30 pm (lens insertion), 10:00 pm (before sleep), 7:00 am (waking), 7:30 am, and 8:00 am (lens removal).

**Results:**

Compared to the lens insertion level, the ET of the lens-wearing eye increased by 5.73% at eye opening (*P* = 0.001). The ET of the non-CL wearing eye and the BT in both eyes did not change after overnight CL wear. Compared to baseline, the CCT of the lens-wearing eye increased by 2.87% upon waking (*P* = 0.003) and recovered 30 min later (*P* = 0.555). In contrast, compared to baseline, the CCT of the non-CL wearing eye did not increase upon waking (*P* = 0.105).

**Conclusions:**

By using UHROCT, we found that overnight CL wear induced different swelling responses in the various sublayers of the cornea.

**Trial registration:**

Retrospectively registered. Registration number: ChiCTR1800015115. Registered 07 March 2018.

Corneal homeostasis provides a refractive media that is essential for vision. Corneal metabolic function is reflected by the thickness of the cornea, which is maintained by the pumping function of the endothelium under normal physiological and diseased conditions [1]. The use of soft contact lenses (CLs) carries an inherent risk of reduced oxygen availability, which can result in edema of the cornea [2–4]. Eye closure results in a dramatically different environment for soft CLs than that experienced with the open eye [2]. Oxygen transmission through the conjunctiva and limbal microvasculature and tear oxygen tension have been shown to decrease during overnight CL wear [5, 6]. Hypoxia associated with corneal swelling may be a potential risk factor to make the cornea susceptible to infection. Schein et al. [7] reported that compared with daily wear, the relative risk of ulcerative keratitis for extended CL wear ranged from 3.90 to 4.21. These findings were reconfirmed by Lim et al. [8] They found that compared with daily use, occasional overnight CL wear presented a 4-times higher risk for microbial keratitis. Therefore, compared to the open eye condition, there is a critical need to monitor corneal thickness with overnight CL wear to limit corneal swelling to a traditionally acceptable extent.

New soft CL technologies and materials have been introduced to improve corneal physiology and alleviate discomfort [2, 9]. Silicone hydrogel CLs increase the supply of oxygen to the cornea as compared to conventional hydrogel lenses. Less dryness and discomfort were observed with silicone hydrogel materials than with conventional hydrogel lenses [10, 11].

Corneal thickness has been analyzed by several different techniques, such as ultrasound, Scheimpflug photography, optical pachometer, confocal microscopy, and time domain optical coherence tomography (OCT) [12–14]. Total corneal swelling was reported after wearing the lenses overnight.^2.13^ Recently, ultra-high resolution OCT (UHROCT) has been used to calculate the thickness of each corneal layer with good repeatability [15, 16]. Hutchings et al. evaluated the change in the thickness of the anterior, stromal, and posterior corneal laminae after eye closure and hydrogel CL wear, and they found that the thickness of the epithelium and stroma was reduced significantly with time [16]. However, subjects in that study had their eyes closed and patched for 3 h after CL insertion; this condition may be different from the environment after overnight CL wear. The aim of the current study was to investigate thickness changes in the corneal epithelium, Bowman’s layer, stroma, and total cornea at the center of the cornea after overnight silicone hydrogel CL wear using UHROCT.

## Subjects and methods

The study was approved by the human subjects review board at the Eye Hospital of Wenzhou Medical University. Each subject signed a consent form and was treated in accordance with the principles of the Declaration of Helsinki. Eleven subjects (6 women and 5 men, mean age: 29.4 ± 2.4 years, range, 27–35 years) without a CL-wearing history were recruited for this study. The eligibility criteria included myopic diopter (D) < − 6.00 D, astigmatism < 2.00 D, and being free of any ocular or systemic disease. All the eligible participants were asked to stay overnight in the research laboratory. The temperature was maintained between 15 °C and 25 °C, and the humidity was kept between 30 and 50% in the examination room.

**Fig. 1:**
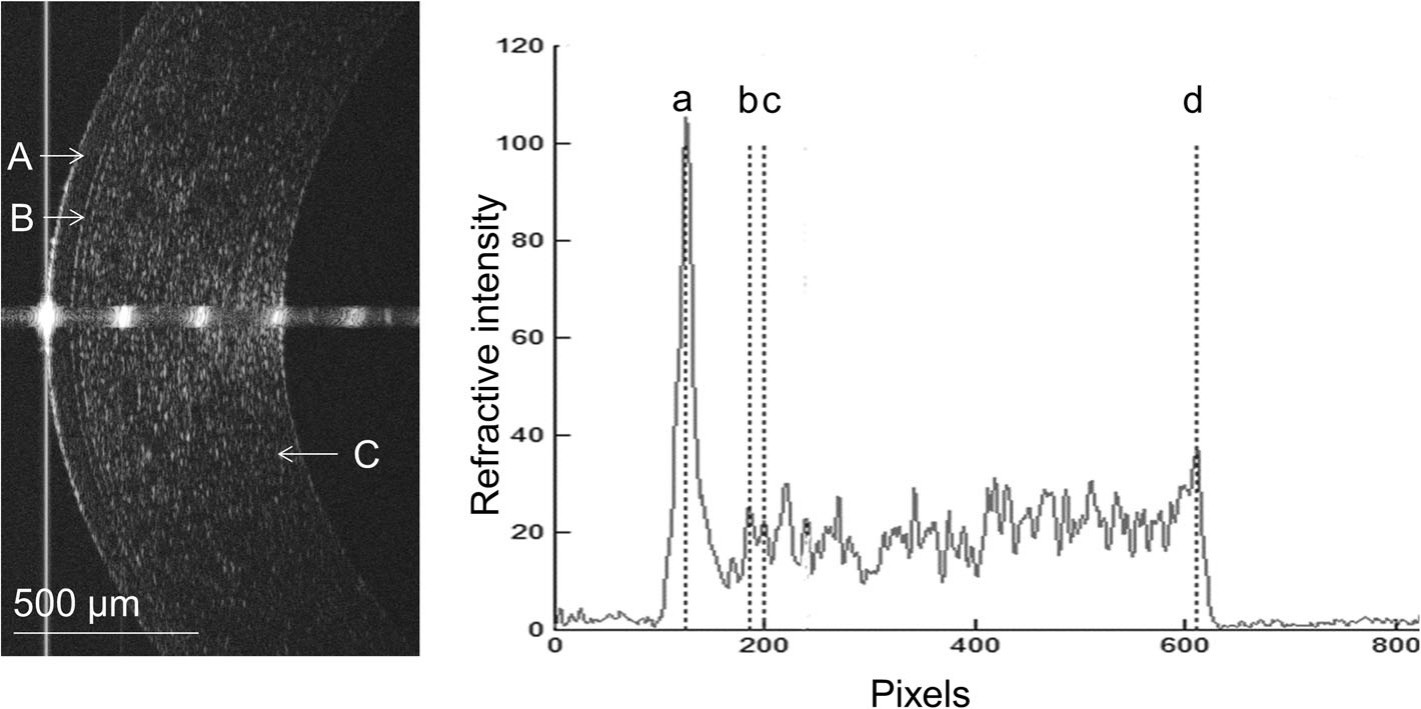
Optical coherence tomography (OCT) image of one 29-year-old subject and longitudinal reflectivity profile. Left: OCT image of the cornea center at baseline. **a**: Epithelium; **b**: Bowman’s layer; **c**: Stroma. Right: Longitudinal reflectivity profile after removing the strong reflectivity at the center. The distance between peaks a and b was the thickness of the epithelium; the distance between peaks b and c was the thickness of Bowman’s layer; the distance between peaks c and d was the thickness of stroma; and the distance between peaks a and d was the total corneal thickness

In this study, a custom-built UHROCT was used to assess the central corneal sublayer thickness. This system has been described in detail in a previous study [15, 17]. In brief, the central wavelength of the light source was 840 nm, and the bandwidth was 100 nm. The actual scan depth was approximately 2.55 mm in air [18], and the scan width was up to 15 mm. The scan speed was 48 frames per second, and the axial resolution was 3 μm. Custom-built software (Matlab, MathWorks, Inc. Natick MA) was used to process images and create OCT longitudinal reflectivity profiles. The boundaries of the corneal sublayers were identified by the peaks of the reflectivity profiles (Fig. 1) [17]. A refractive index of 1.40 was used for each layer, and the refractive index of 1.389 was used for the total corneal thickness when translating from optical length to physical length. Three images were taken, and an average thickness was obtained.

**Fig. 2:**
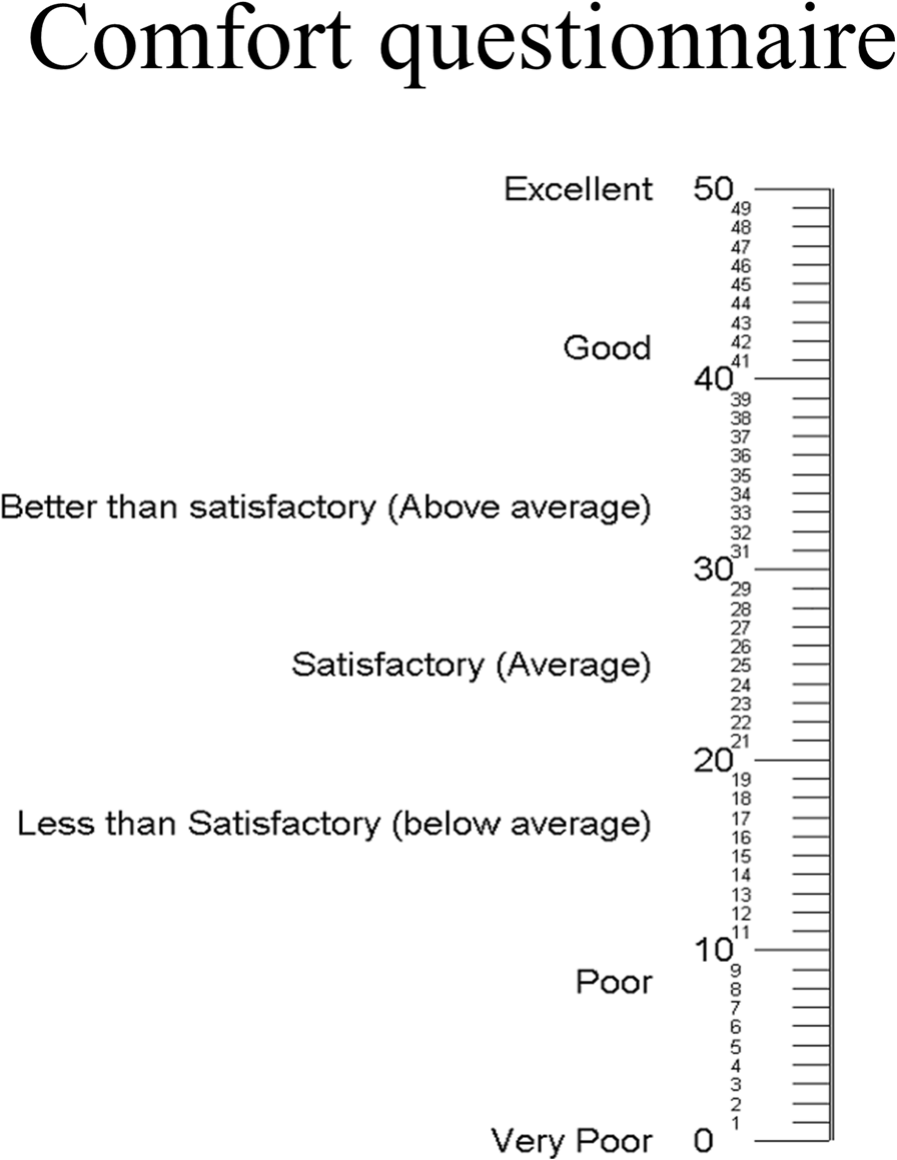
A questionnaire was used to rate ocular comfort. Very poor comfort was defined as 0, and excellent comfort was defined as 50

One soft CL (PureVision; Bausch & Lomb, Rochester, NY) was inserted into the right eye of each subject, and the left eye was used as a control. The base curve of the CL was 8.6 mm, with a spherical diopter of − 3.00 D. The lens was inserted at 9:30 pm and removed at 8:00 am the next morning. UHROCT was performed at 9:00 pm (baseline), 9:30 pm (lens insertion), 10:00 pm (before sleep), 7:00 am (waking), 7:30 am, and 8:00 am (lens removal). Ocular comfort was evaluated by a questionnaire with a continuous scale from 0 to 50 [19]. Very poor comfort was defined as 0, and excellent comfort was defined as 50 (Fig. 2). Ocular comfort was rated at each time point.

Data analysis was conducted with Statistical Package for the Social Sciences software (SPSS, version 13.0; SPSS, Cary, NC). On the basis of the normal epithelial thickness of 52.5 ± 2.4 [17], the minimum sample size to detect a 2.0 group difference with a 90% statistical power was 5 [20]. Considering the normal Bowman’s layer thickness of 17.7 ± 1.6 [17], the minimum sample size to detect a 0.8 group difference with a 90% statistical power was 10 [20]. On the basis of normal stromal thickness of 459.1 ± 26.8 [17], the minimum sample size to detect a 15.0 group difference with a 90% statistical power was 8 [20]. By considering the normal total corneal thickness of 529.4 ± 27.1 [17], the minimum sample size to detect a 15.0 group difference with a 90% statistical power was 8 [20]. Thus, 11 cases in this study were adequate. Repeated-measures analysis of variance was used for overall statistical testing. Bonferroni post-hoc tests were used to determine whether there were differences in corneal thickness among the different visits. Adjusted *P* values less than 0.0083 were considered significant. Data are presented as mean ± standard deviation (SD).

## Results

**Fig. 3:**
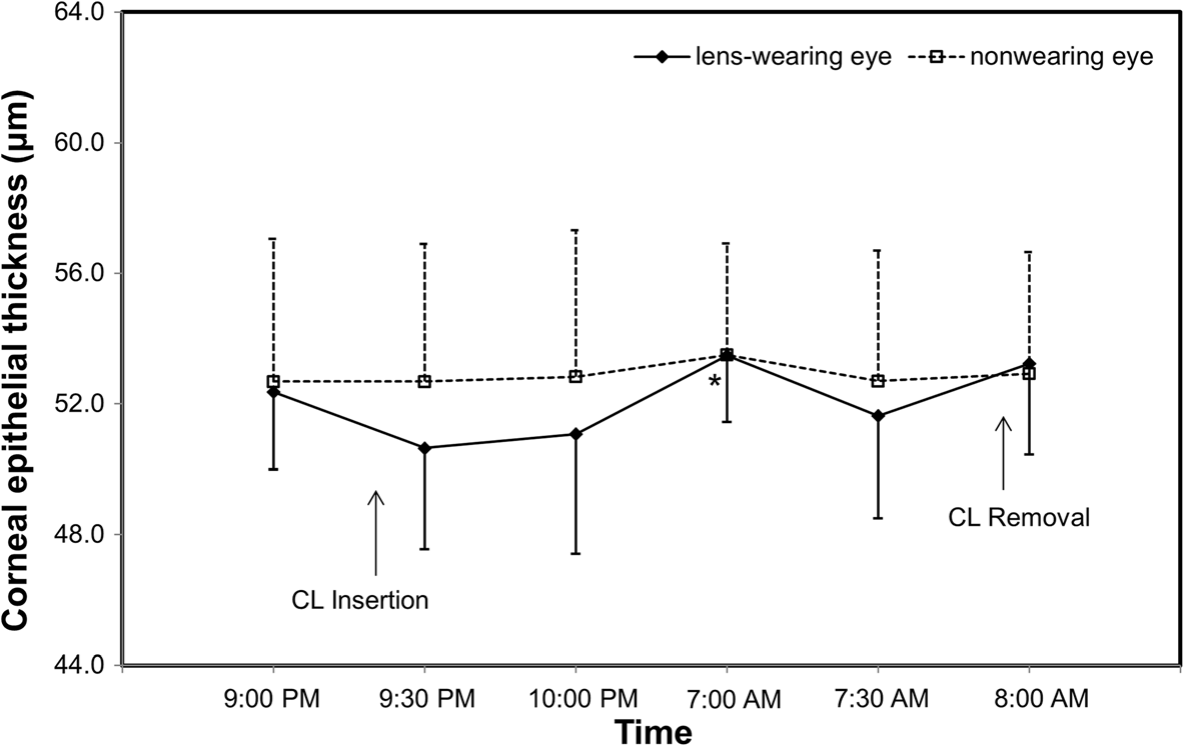
Corneal epithelial thickness after overnight contact lens (CL) wear. Compared to the lens insertion level, the epithelial thickness of the lens-wearing eye increased at eye opening (*P* = 0.001). The epithelial thickness of the non-CL-wearing left eye did not change at waking (*P* > 0.05).*: *P* < 0.05, compared with the lens insertion level

**Fig. 4:**
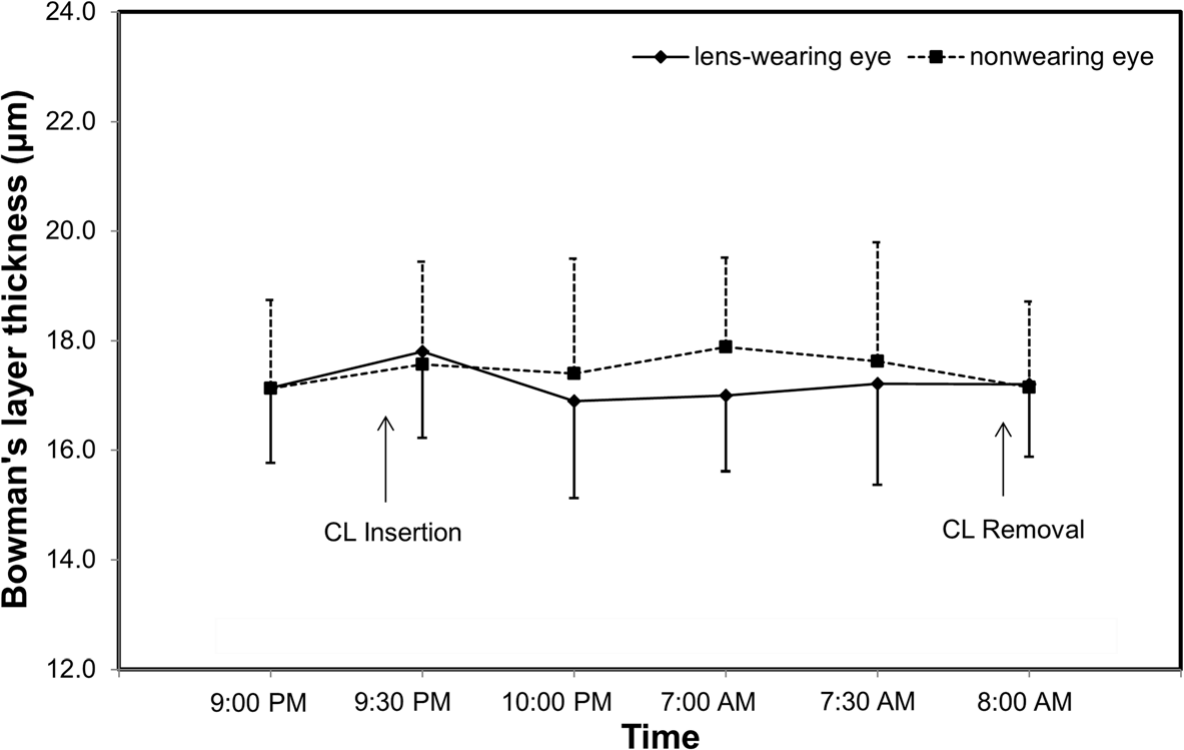
Bowman’s layer thickness after overnight contact lens (CL) wear. The thickness of Bowman’s layer in both eyes did not change significantly after overnight CL wear (P > 0.05)

**Fig. 5:**
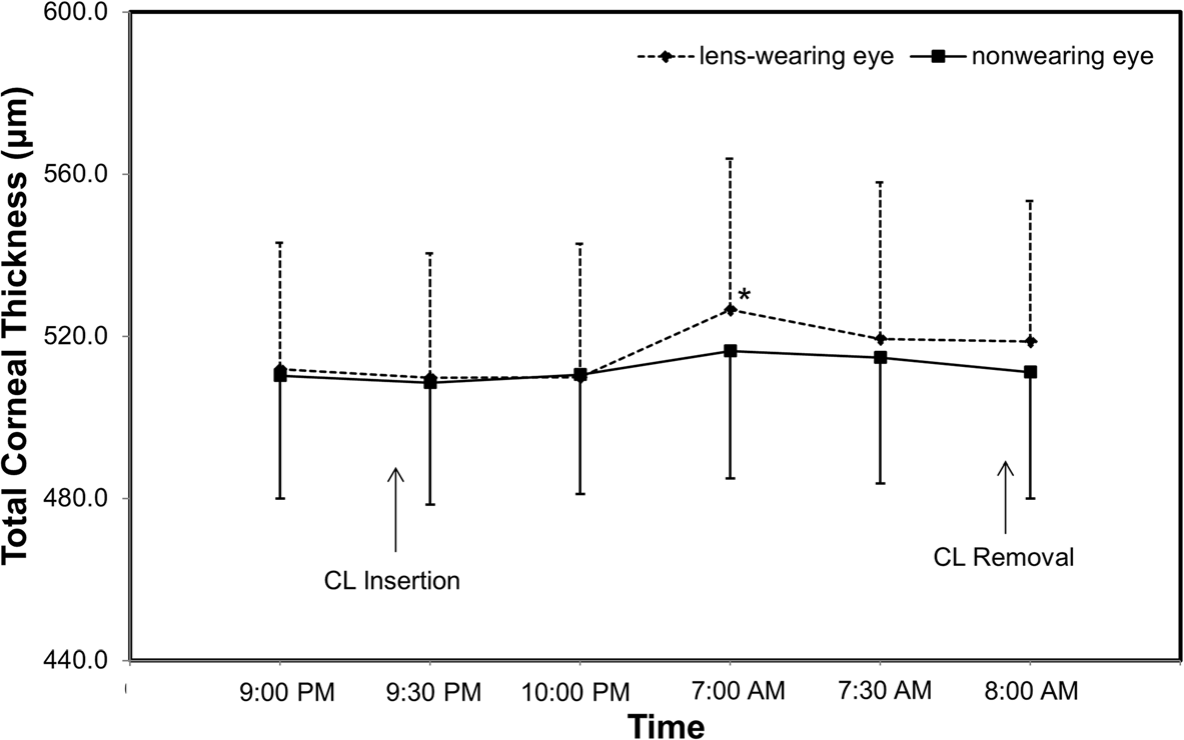
Total corneal thickness after overnight contact lens (CL) wear. Compared to baseline, the total corneal thickness of the lens-wearing right eye increased at waking the next day (*P* = 0.003) and recovered 30 min later (*P* = 0.555). In contrast, the total corneal thickness of the non-CL wearing left eye did not increase at waking (*P* = 0.105). *: P < 0.05, compared with baseline

**Fig. 6:**
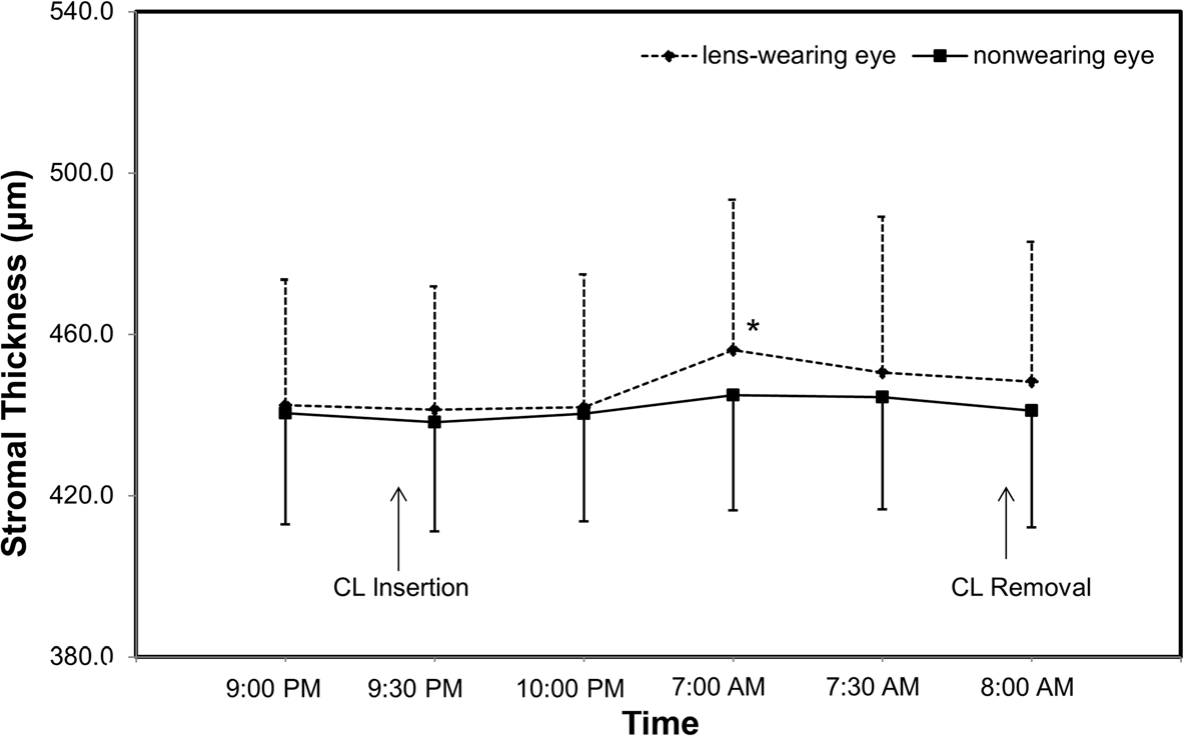
Stromal thickness after overnight contact lens (CL) wear. Compared to baseline, the stromal thickness of the lens-wearing right eye increased at waking (*P* = 0.005) and recovered 30 min later (*P* = 0.383). In contrast, the stromal thickness of the non-CL-wearing left eye did not increase at waking (*P* = 0.467). *: P < 0.05, compared with baseline

**Fig. 7:**
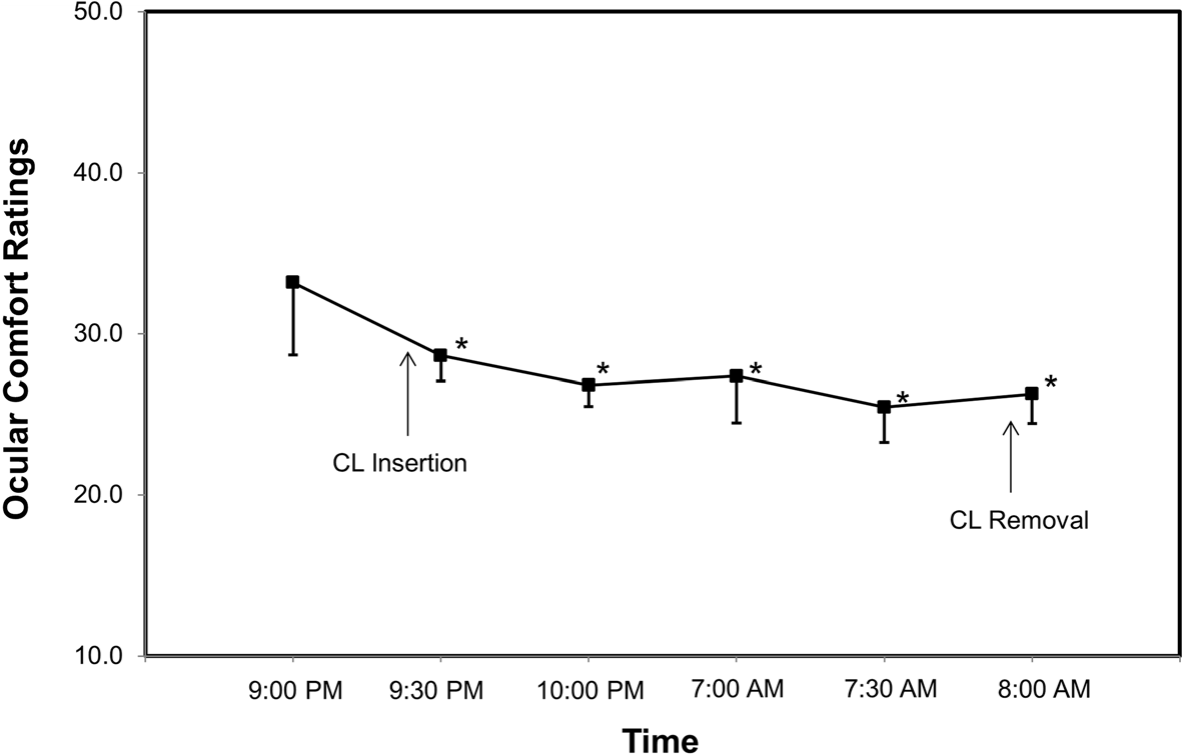
Ocular comfort ratings after overnight contact lens (CL) wear. Ocular comfort decreased after lens insertion (*P* = 0.007) and remained low during CL wear. *: P < 0.05, compared with baseline

The average epithelial thickness of the lens-wearing right eye was 52.4 ± 2.4 μm at baseline. The epithelial thickness decreased after lens insertion (50.6 ± 3.1 μm), but this decrease was not statistically significant (*P* = 0.027, Fig. 3). Compared to the lens insertion level, the epithelial thickness increased to 53.5 ± 2.0 μm at eye opening (*P* = 0.001, Fig. 3). The epithelial thickness of the non-lens-wearing left eye did not change at waking (*P* > 0.05, Fig. 3). The thickness of Bowman’s layer in both eyes did not change significantly after overnight CL wear (P > 0.05, Fig. 4). Compared to baseline, the total corneal thickness of the lens-wearing right eye increased by 2.87% upon waking the next day (*P* = 0.003, Fig. 5) and recovered to baseline 30 min later (*P* = 0.555, Fig. 5). In contrast, compared to baseline, the total corneal thickness of the non-lens-wearing left eye did not increase at waking (*P* = 0.105, Fig. 5). The trend of stromal thickness was similar to that of total corneal thickness (Fig. 6). Ocular comfort decreased after lens insertion (*P* = 0.007, Fig. 7) and remained lower during CL wear (Fig. 7).

## Discussion

The fit of soft CLs in vivo has been subjectively assessed by slit-lamp biomicroscopy in the clinic or quantified by video capture, but both methods provide limited resolution [21, 22]. Shen et al. [23] characterized the conjunctival buildup and tear film gaps of differently designed CLs; however, the clinical significance of these new parameters remains unclear. Hall et al. analyzed the corneal-scleral shape profile to predict the CL fit [24]. Our previous studies evaluated the entire CL thickness by using long scan depth OCT based on a complementary metal oxide semiconductor and a charge-coupled device camera [25, 26]. The axial resolution was compromised with a long scan depth, and an ultra-high resolution (≤ 5 μm) did not succeed in extending the scan depth OCT. Therefore, the thickness of the corneal sublayers was not analyzed in these previous studies [25, 26]. Recently, UHROCT has been demonstrated to be a feasible method of measuring the sublayer thickness of the cornea in vivo. In this study, we focused on thicknesses of separate layers of the cornea after overnight CL wear.

By using UHROCT, the epithelial and total corneal thickness at baseline in the present study was similar to that of our previous studies [15, 17]. Fonn et al. [13] used an optical pachometer,and observed that the total corneal thickness at baseline was 540 μm, which was in agreement with that observed in the present study. In contrast, by using time domain OCT, Wang et al. [27] observed that the measured epithelial thickness was 59.9 μm, which was thicker than that of the present study. By using UHROCT, Hutching et al. [16] noted that the mean epithelial thickness and total corneal thickness of 8 non-CL wearers was 58.40 μm and 613.91 μm, respectively, which were much higher than the values obtained in this study. Reasons for the discrepancy between these studies may be explained as follows: First, the demographic characteristics of the subjects of these studies, including age and ethnicity, were different. Second, instruments with different resolutions were used to acquire corneal images, and some studies used in-house-developed software for analysis. The refractive index used to convert the optical measurement to the physical measurement in these programs may be different, which may lead to overestimation or underestimation of the corneal thickness of each layer.

By using an optical pachometer, the central corneal swelling induced by the lotrafilcon A lens was found to be 2.71% [13]. Moezzi et al. used the same instrument,and observed that the percentage of central corneal swelling with the comfilcon A and lotrafilcon A lenses was 4.1 and 4.0%, respectively, for an 8-h overnight period [28]. We found the corneal swelling to be 2.87% after overnight CL wear with a material of balafilcon A; this magnitude of swelling was similar to that noted in previous studies using silicone hydrogel lenses. In contrast, as measured with a Holden-Payor pachometer, corneal swelling was found to be approximately 7.0% in symptomatic and asymptomatic CL wearers who were wearing bilateral disposable etafilcon A lenses overnight [29]. After 3 h of hydrogel CL wear with eye closure, the total corneal swelling was approximately 8.3% with UHROCT [16] and 13.4% with time-domain OCT. [14] Silicone hydrogel CLs with high oxygen transmissibility were developed to relieve hypoxia during overnight CL wear [2]; this may be the main reason that corneal health was better with silicone hydrogel than with hydrogel. In addition, corneal swelling induced by patching with eye closure for 3 h may be different from that induced by overnight CL wear.

The response to overnight CL wear was different in the various sublayers of the cornea. The epithelial thickness had a decreased trend immediately after lens insertion; this may be caused by the lens exerting mechanical pressure on the ocular surface. After overnight CL wear, at eye opening, corneal epithelial thickness increased by 5.73%. The thickness of Bowman’s layer did not change after 9 h of sleeping with the lenses overnight. Thus, the increase in the central corneal thickness was mainly due to the increase in the epithelial and stromal thicknesses. The swelling of the epithelial layer in the present study was similar to the swelling found after hydrogel CL wear with eye closure [16]. Compared to the stroma, the limited expansion capacity of the epithelium may have contributed to the similar expansion found in both studies. The stronger regions of the cornea are locked anteriorly and have been proposed to be less affected than the posterior sublayer when the cornea swells [16, 30]. As we expected, the epithelium showed less swelling than the posterior lamellae. Furthermore, the stroma recovered to baseline levels within 30 min of eye opening. The corneal epithelium and endothelium are two barriers to maintain corneal transparency and hydration. Thirty minutes after the eye opening, the increased supply of oxygen to the eye led to a decrease in corneal edema and recovery of the normal corneal structure.

The response of the lens-wearing right eye was different from that of the non-lens-wearing left eye after overnight eye closure. In the present study, both CL wear and overnight eye closure induced hypoxia in the right eye, whereas the response of the left eye was mainly due to overnight eye closure. Therefore, the epithelium and stroma of the left eye did not change after overnight eye closure. As reported by Fonn et al., the corneal swelling of the lens-wearing eye can affect the swelling of the contralateral control eye [13]. Because of the possible existence of a sympathetic physiological response, the use of the contralateral non-lens-wearing eye as the control eye, as done in this study, may differ from using both eyes without lens wear as the control.

This study had some limitations. First, we focused on the corneal sublayer changes at the center of the eye in the present study. Further studies on the superior, inferior, nasal, and temporal limbus changes after overnight CL wear need to be conducted. Second, only one soft CL with the same base curve was used for all subjects in the present study. Because the ocular surface character of each subject was different, the lens fit may have been different, although all fits are acceptable in the clinic [31]. The corneal sublayer response to CLs with different lens materials, different lens designs, or different base curves needs to be investigated in future studies. Third, changes in corneal sublayer thickness after only 1 night of soft CL wear was investigated. However, further studies with longer period of extended wear are needed to better understand the corneal effect of hypoxia after hydrogel CL and silicone hydrogel CL use.

## Conclusions

In summary, by using UHROCT, we found that the swelling response induced by overnight CL wear was different in the various sublayers of the cornea.

## Abbreviations

BT: Bowman’s layer thickness
CCT: central corneal thickness
CLs: contact lenses
D: diopter
ET: epithelial thickness
OCT: optical coherence tomography
SD: standard deviation
ST: stromal thickness
UHROCT: ultra-high resolution OCT
